# Mucus-derived exosome-like vesicles from the Spanish slug (*Arion vulgaris*): taking advantage of invasive pest species in biotechnology

**DOI:** 10.1038/s41598-022-26335-3

**Published:** 2022-12-16

**Authors:** Michaela Liegertová, Alena Semerádtová, Michaela Kocholatá, Michaela Průšová, Lenka Němcová, Marcel Štofik, Sylvie Kříženecká, Jan Malý, Olga Janoušková

**Affiliations:** 1 Centre of Nanomaterials and Biotechnology, Faculty of Science, Jan Evangelista Purkyně University in Ústí nad Labem, Ústí nad Labem, Czech Republic; 2 Department of Biology, Faculty of Science, Jan Evangelista Purkyně University in Ústí nad Labem, Ústí nad Labem, Czech Republic; 3 Department of Environmental Chemistry and Technology, Faculty of Environment, Jan Evangelista Purkyně University in Ústí nad Labem, Ústí nad Labem, Czech Republic

**Keywords:** Biotechnology, Molecular biology, Zoology

## Abstract

The slug *Arion vulgaris* has attracted major attention as one of the worst invasive herbivore pests in Europe and is renowned for the stiff mucus it secretes for locomotion. In this study we focused on the isolation and characterisation of extracellular vesicles, specifically exosomes and exosome-like vesicles, from Arion secretions. We developed a method for slug mucus collection and subsequent vesicle isolation by ultracentrifugation. The isolated vesicles with an average diameter of ~ 100 nm carry abundant proteins and short RNAs, as well as adhesion molecules similar to mammalian galectins. We demonstrated that the slug extracellular vesicles are internalised by plant cells and human cancer cells in in vitro assays and are loadable by bioactive compounds, which makes them an interesting tool for utilisation in biotechnology.

## Introduction

Spanish slugs (*Arion vulgaris*, Moquin-Tandon, 1855) are the most common European gastropod species listed among the worst invasive pests in Europe. Arion slugs are voracious herbivores known to cause substantial ecological^1–5^ and economical damage^6,7^. Additionally, they act as vectors for pathogenic bacteria and as hosts for parasites, which harm domestic pets and cattle^8–10^. *Arion vulgaris* produces highly viscous, sticky and hard to remove ventral mucus generated mostly by five subepithelial laterally and ventrally located gland types which enables it to overcome many types of surfaces and natural as well as artificial obstacles which might contribute to the success of their geographical dispersal and the level of current infestation across Europe^11–13^.

In this study we decided to search for extracellular vesicles, specifically exosomes and exosome-like vesicles (EXs) in the Arion mucus. EXs are extracellular vesicles with an average diameter of ~ 100 nm that are generated in the endosomal compartment of most eukaryotic cells with a huge potential in biomedical applications (as thoroughly reviewed in^14,15^). For example, doxorubicin (DOX, Adriamycin) isolated from *Streptomyces *sp. frequently used as the first-line therapy for a variety of solid malignancies (approved by the FDA in 1974) is known for its dose-dependent cardiac and severe systemic toxicity, which could be significantly reduced by conjugation with exosomes derived from human in vitro cell cultures^16^.

Next to mammals being the traditional source of EXs for research and recent therapies the non-mammalian sources of EXs are recently gaining in popularity for their interesting properties and complex roles (ranging from inter-species interactions to inter-kingdom communication)^17–22^. EXs from diverse sources like the bee products, snake venom or plants and fruits are becoming an increasingly compelling toolbox for the agricultural, pharmaceutical and biomedical industry^17,20,23,24^. Inspired by the progress in this research field (and by the availability of the Arion slugs for potential large scale EXs isolation) we set out to inspect the pestiferous *Arion vulgaris* as a potential alternative source of EXs for applications in biotechnology**.**

In this study, we provided a simple and efficient method for slug mucus collection and subsequent EXs isolation by ultracentrifugation. The slug EXs were characterised and visualised by Nanoparticle size analysis and transmission electron microscopy, along with the size distribution profile provided by the Dynamic light scattering technique. The EXs protein content was quantified by the bicinchoninic acid and CBQCA protein quantitation assays, RNA isolation was performed, and the presence of adhesion molecules was verified using western blotting. Additionally, the model drug loading efficacy of the slug EXs along with cell uptake in in vitro conditions were demonstrated.

## Results

**Figure 1:**
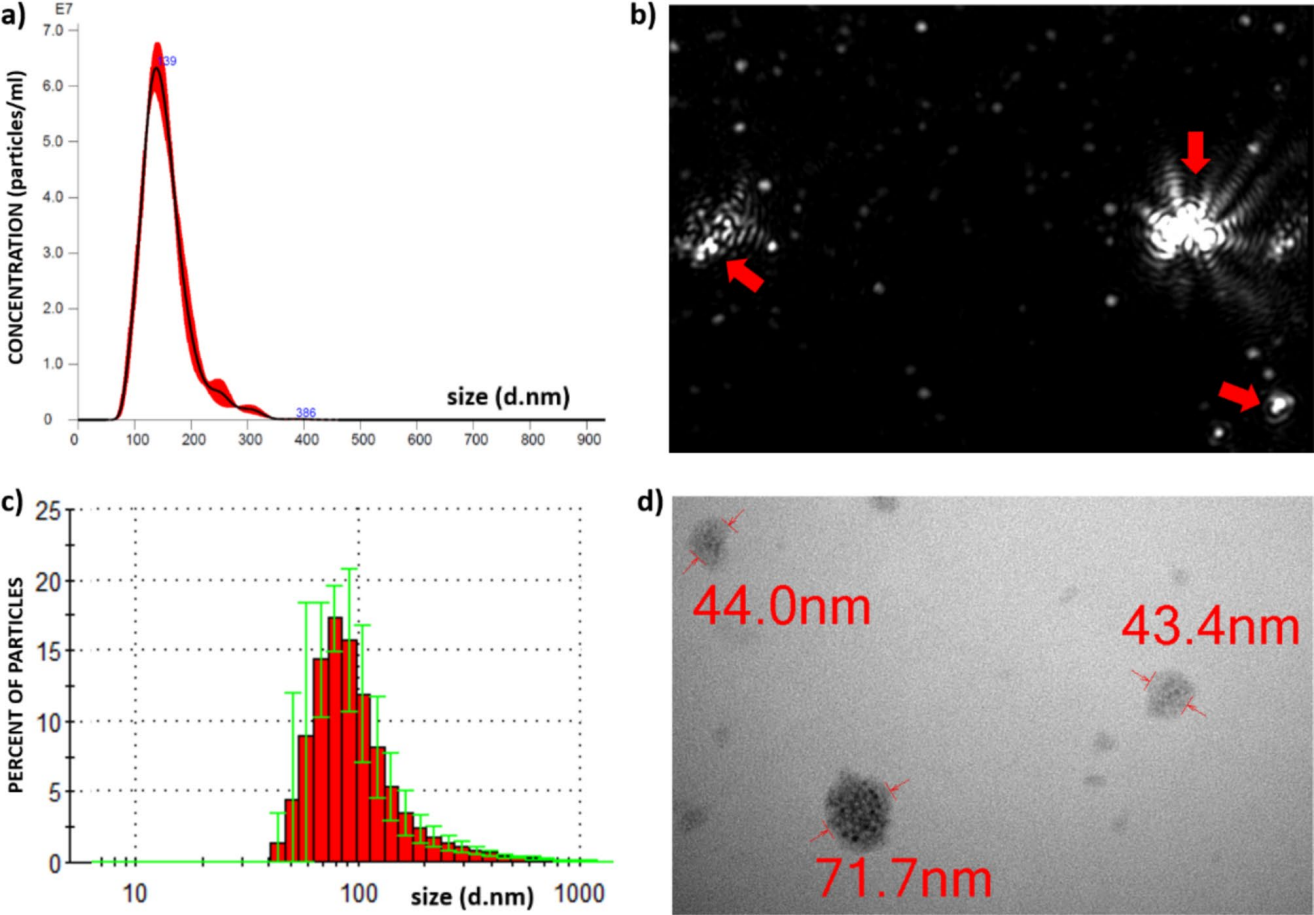
Slug EXs characterisation. (**a**) Representative histogram illustrating the nanoparticle size distribution of slug EXs in liquid suspension as determined by Nanoparticle tracking analysis. (**b**) Visualisation of the individual EXs (single white dots of various sizes) and their aggregates (red arrows) from a video recording of the particles moving under Brownian motion. (**c**) Representative histogram illustrating the size distribution of slug EXs as determined by Dynamic light scattering analysis. (**d**) Transmission electron micrographs of slug EXs showing vesicular structures.

The presence of EXs in the mucus collected from *Arion vulgaris* slugs was verified using Nanoparticle tracking analysis (NTA), Dynamic light scattering (DLS) and Transmission electron microscopy (TEM) as shown in (Fig. 1a–d). The concentration of slug EXs in 100× diluted isolate was 5.27 × 10^9^ (± 6.65 × 10^7^) particles/ml with a mean diameter of 154.2 nm (± 43.4 nm) as determined by NTA. The EXs tend to form aggregates, as demonstrated by analysis of a video record of the particles moving under Brownian motion. As shown by DLS, 90% of the EXs cluster within the size range of 40–160 nm. TEM analysis confirmed the presence of round shaped vesicles of various diameters ranging from 44 to 160 nm.

**Figure 2:**
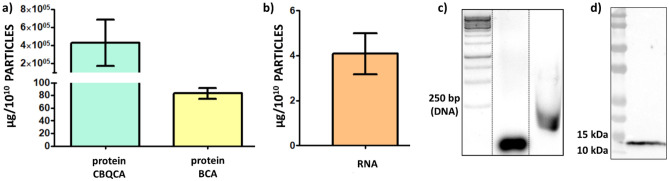
Slug EXs content analysis. (**a**) Comparison of the EXs protein concentrations provided by BCA and CBQCA quantification assays. (**b**) RNA content of the slug EXs. (**c**) RNA isolated from the slug EXs (lane 3) compared to loading control siRNA (21mer duplex with dTdT overhang, Sigma-Aldrich; lane 2) and to the DNA ladder (lowest band = 250 bp; lane 1). (**d**) Western blot analysis of slug EXs lysate using a Galectin-1/LGALS1 primary antibody against human galectin-1 protein (for the uncropped western blots and gels see supplemental data).

The average protein concentration in the slug EXs lysate was 108.71 μg (± 4.79 μg) per 10^10^ particles according to BCA protein assay as compared to the average protein content of 429.32 mg (± 210.49 mg) per 10^10^ particles, when quantified using the CBQCA protein assay (Fig. 2a). Since the BCA, one of the most widely used methods for quantifying exosomes and their protein content in the literature, could produce erroneous values when common membrane lipids/phospholipids are present^25,26^, combining the NTA and CBQCA protein assay should be the preferred methods for the slug EXs and their protein quantification.

The slug EXs contained 4.09 μg (± 0.91 μg) RNA per 10^10^ particles (Fig. 2b). The main fraction of RNA isolated from the slug EXs consisted of abundant short RNAs with an approximate size < 250 bases (Fig. 2c). Using western blotting with primary antibodies against exosomal markers and adhesion molecules—lectins (galectin-1, 3, 8, 9) known to be present in human EXs we were able to identify one of the slug EXs proteins as galectin-1-like protein (Fig. 2d), as shown by the band of size between 14 and 15 kDa.

**Figure 3:**
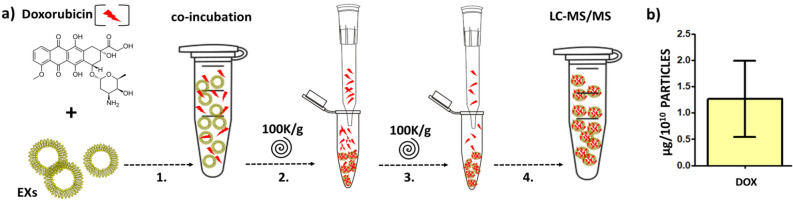
Slug EXs loading with DOX. (**a**) After co-incubation of DOX with EXs for 2 h at 37 °C, (1) two subsequent washing steps were applied to remove the residual unbound DOX. The mixture was ultracentrifuged for 30 min at 100,000×*g*, the supernatant containing the free DOX was discarded, the pellet containing the nanovesicles washed with PBS, resuspended in 1 ml of PBS, and this step was repeated (2.–3.) before quantification of the EXs-bound DOX by LC–MS/MS (4). (**b**) Resulting uptake of the DOX per 10^10^.

The doxorubicin (DOX) loading capacity by slug EXs was analysed using Liquid chromatography with Tandem mass spectrometry (LC–MS/MS), confirming successful uptake of 1.26 µg (± 0.59 µg) of the precursor ion doxorubicin per 10^10^ particles. The estimated EXs loading efficacy of approximately 2.5% was calculated by comparing the initial entry concentration of DOX to the final DOX-EXs concentration (100 µg/ml DOX vs. 2.5 ± 0.27 µg/ml DOX-EXs) after the co-incubation and subsequent washing steps (Fig. 3a,b).

**Figure 4:**
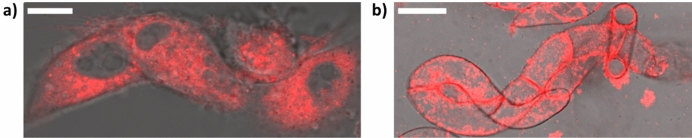
Slug EXs cell uptake assays. (**a**) Laser scanning confocal microscopy analysis of the U87 human glioblastoma cells after the BODIPY TR Ceramide stained EXs uptake assay. Scale bar = 10 µm. (**b**) Confocal microscopy analysis of the BY-2 plant cells after the EXs uptake assay. Scale bar = 50 µm.

The in vitro cell internalisation assay of slug EXs stained with BODIPY TR Ceramide confirmed the uptake of EXs by U87 human glioblastoma and BY-2 plant cells, as demonstrated by confocal microscopy imaging (Fig. 4a,b). After the internalisation of the stained EXs, the fluorescent dye was clearly detectable within the cytoplasm of both human and plant cell lines.

## Discussion

Mucus from terrestrial gastropods (snails and slugs) is a complex substance generally secreted by epidermal glands that cover the external surface of the animals and has various functions and properties (moisturising, lubricating, adhesive, reparative, protective, defensive, etc.)^27–29^. Its composition may vary across species and based on its function^30,31^, but generally comprises of over 90% water and a variety of proteoglycans, glycosaminoglycans, glycoprotein enzymes, hyaluronic acid, copper peptides, antimicrobial, antifungal, and antiviral peptides^31,32^, and metal ions^33–35^. Slugs lacking the protective shell of snails are known to secrete an extremely stiff mucus when endangered or disturbed by predators or humans^36–38^.

Snail and slug secretions were shown to be exceptionally promising substances for use in biomedicine and the cosmetic industry, as well as for industrial applications^12,39–45^. The mucus contains proteins like collagen, elastin, glycolic acid (capable of penetrating skin and causing increased collagen synthesis^46^) and allantoin (known for its promotion of wound healing and cell proliferation^47,48^). Snail mucus shows potential for regenerating and repairing bone and teeth^49^. Inspired by these studies, we focused on inspecting the Arion mucus for the presence of EXs, which were proposed to be a common component of animal secretions^18^. The isolated slug EXs show a size distribution similar to mammalian/human sources and carry typical cargo such as proteins and short RNAs.

Most of the attention in the EXs research field has been focused on human (mammalian) EXs sources, providing a basic understanding of their role in biological processes and investigating their potential use in biomedicine. However, there is a growing interest in the less conventional sources, ranging from invertebrates to plants and fungi^17,50–52^. EXs are not restricted only to communication within an individual or within certain species but have been repeatedly shown to provide inter-species as well as inter-kingdom interactions^18^. Aside from their important role in cell-to-cell communication (acting as vehicles for specific molecules such as proteins, enzymes, growth factors or RNAs), EXs were shown to represent a general mechanism for mediating communication within host-parasitic interactions. They are known to act as carriers of virulence factors and various effector proteins from parasites to their hosts. Consequently, EXs are of particular interest to biomedicine since they mediate and modulate pathogenic responses and processes via modifying the host's gene expression and response of the host's immune system^21^. Nevertheless, an infection (or infestation in the case of slugs) has a bidirectional effect between the parasite and its host, and EXs are accordingly produced as a response to parasites. Plant EXs were shown to participate in communication between plants and their pathogens by the transportation of various proteins and small RNAs. During the fungal infection of the plant, small RNAs such as miR166 and miR159 are transported via plant EXs into the hyphae of the fungus, where they regulate the expression of virulence enzymes^53^. However, the transport of biomolecules can be bidirectional, as small RNAs of the fungus can target plant defence genes^54^.

Our data support the hypothesis of a possibly complex reciprocal interplay by the EXs in the role of response mediators between the Arion herbivore and its plant diet. The slug EXs are loaded with proteins and short RNAs and were shown to be internalised by plant cells in in vitro conditions (Fig. 4a,b). Plants are known to respond to herbivory similarly to infections, using various biochemical and molecular mechanisms to counterattack the feeding organism. Some of the compounds generated by plants are defensive substances affecting the processes of feeding, growth, and survival of the herbivore^55,56^. In response, the voracious *Arion vulgaris* might use EXs to modify the plant's stress response in its favor. Another possible role of slug EXs is the protection of the slugs against infection by bacteria or viruses as well as against their potential predators or in mediation of communication with other Arion slugs or terrestrial gastropods via the mucus trails^29,57–60^. The potential of slug EXs as a tool for plant modification in plant biotechnology or as a tool for gastropod pest control in agriculture may be worth investigating.

The EXs are becoming increasingly popular in the field of biomedicine. Most of the ongoing exosome-focused clinical trials concentrate on EXs of human origin as diagnostic or prognostic biomarkers (i.e.**,** for many types of cancers). However, several trials are already focused on the use of EXs as therapeutic agents in various types of diseases (as recently reviewed in^61^). EXs as a tool for cell delivery applications show reduced immunogenicity when compared to viral vectors, steadiness in the bloodstream, and enhanced absorption potential when compared to liposomes, and have the ability to overcome physiological barriers (i.e.**,** blood–brain barrier)^62^. Since the workflow for human EXs characterisation was already established in our lab (i.e., panel of antibodies against exosomal adhesion molecules and markers of human origin) we decided to use our set of primary antibodies against human exosomal adhesion molecules (lectins) to search for related molecules in the slug EXs. The positive signal for human galectin-1 antibody in the slug EXs revealed the presence of human galectin-1-like proteins. Galectins are a family of soluble, non-glycosylated lectins of small molecular weight between 14 and 39 kDa that bind to galactose- and N-acetyllactosamine-based motifs that are widely conserved across species and have been linked to exosome uptake by target cells^63,64^. In humans, galectin-1 is attached to the surface of exosomes derived from placental mesenchymal stromal cells, tumour or syncytiotrophoblast cells and is involved in exosome adhesion^65–67^. Moreover, it was shown that the uptake of galectin-1 enriched EXs by human cancer cells leads to the upregulation of its intracellular concentration, which strongly affects cancer cell migration^68^. According to our data (Fig. 2d), galectin-1-like protein is present in the slug EXs, possibly serving an analogous function in EXs adhesion and uptake, suggesting that slug EXs could indeed serve as an efficient vehicle for bioactive molecules into human cancer cells in cell delivery**-**based therapies.

DOX is one of the most widely used cancerostatics for treating a multitude of cancers. It induces cell death through multiple intracellular targets: reactive oxygen species generation, DNA adduct formation, topoisomerase II inhibition, histone eviction, or Ca^2+^ and iron homeostasis regulation^16^. One of the numerous drawbacks of DOX is its dose-dependent cardiac and severe systemic toxicity^69^. The encapsulation of DOX into nanoscale particle drug delivery systems was shown to offer advantageous biodistribution, pharmacokinetics, and controlled release while lowering its systemic toxicity^70^. In the past two decades, several nanotechnology-based DOX conjugation systems have been developed, some of which have received FDA approval^16^. Particularly, exosomes are known for their ability to cross the blood–brain barrier and to be internalised into certain brain cells (such as glioma cells). The delivery of DOX to glioblastoma cells via exosomes was shown to substantially inhibit tumour growth in a zebrafish model^71^.

Several loading methods were used in exosome loading experiments, with direct co-incubation being the most studied procedure due to its simplicity and lack of notable impacts on exosome structure and content^72^. Soluble substances such as DOX cannot be precipitated after centrifugation and may be discarded in the ultracentrifuge supernatant^71,73^, thus the most prevalent strategy for isolating residual free DOX from loaded EXs is centrifugation-based separation^72^. In this regard, we performed a simple proof-of-concept experiment to verify the potential of the slug EXs for cell delivery applications and have shown that slug EXs are loadable by bioactive compounds, as was confirmed by a DOX loading assay by passive cargo loading (direct co-incubation of EXs with DOX). The relatively low loading capacity of slug EXs (approximately 2.5%) would be sufficient for the use in human therapies, but the efficacy could be significantly enhanced by using active cargo loading approaches (i.e., electroporation or sonication).

Finally, as proved by confocal microscopy (Fig. 4a,b), the slug EXs were shown to be internalised by human glioblastoma cells, which serve as an in vitro model cell delivery system for EXs from conventional sources^74^.

So far, human mesenchymal stem cells or human dendritic cells are the main sources for therapeutic EXs^75^. Cultivating these cell cultures in in vitro conditions in sufficient quantities for EXs harvesting is laborious and economically demanding (i.e.**,** high quality cell culture media consumption), which makes the search for cheaper and abundantly available alternatives extremely appealing. Taken together with the low demands on culturing the Arion slugs in laboratory conditions, the vast ecological and economic damage caused by this "super-villain" could be at least partly compensated by providing an interesting tool for biotechnology and biomedicine in the near future.

## Methods

### *Arion vulgaris* collection

2 kg of Arion slugs were collected in private gardens during summer seasons in 2021 and 2022 in north-west Bohemia (Czech Republic). Prior to mucus collection, slugs were kept in plastic cages or glass aquariums filled with garden soil and wood debris and were fed with fresh green salad and chopped cucumbers ad libitum for at least 48 h to fully adapt to the artificial laboratory conditions. After the mucus collection process, the slugs were released back to their natural habitat. Species identification was carried out by the Biology Department of Jan Evangelista Purkyně University in Ústí and Labem.

### Mucus collection and EXs isolation

**Figure 5:**
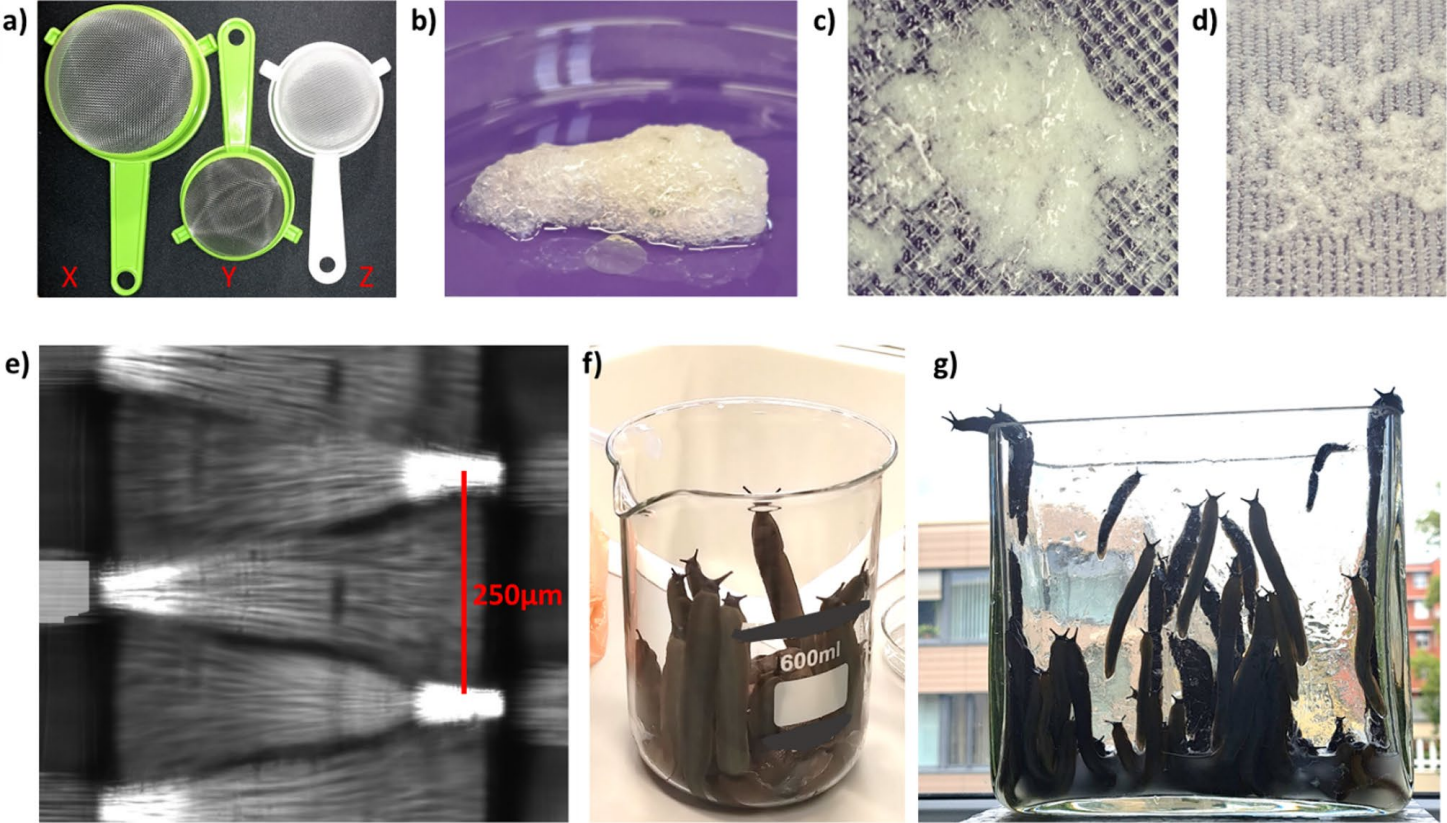
Mucus collection procedure. (**a**) Commonly available kitchen sieves with decreasing mesh size (X = 1 × 1 mm, Y = 0.75 × 0,75 mm, Z = tightly woven filaments) were used to filter the stiff and insoluble fraction of the mucus. (**b**) Fraction of the mucus left after filtration with X; Y = (**c**); Z = (**d**). (**e**) Detail of the Z’s mesh filaments under microscope (however, any sieve with mesh size of 0.5 × 0.5 mm down to 0.2 × 0.2 mm could be used as effectively). (**f**,**g**) After incubation of the slugs in the PBS buffer the slugs spontaneously crawl out of the containers to be collected, rinsed gently with tap water, and left to recover.

Prior to mucus collection each slug was thoroughly rinsed under tap water to get rid of the attached debris and faeces, followed by a short rinsing with deionized water. 250–300 g of rinsed slugs (20–25 individuals) were transferred to 600 ml beakers filled with 50 ml of 0.01× Phosphate-buffered saline + 0.0027 M KCl + 0.137 M NaCl pH 7.4 (PBS) (mucus solution collected from two beakers was pooled together for subsequent steps) or 600–750 g of slugs (approximately 50 individuals) were transferred to 2 l glass container filled with 100 ml PBS (Fig. 5f,g). Only mature and fully active animals within the weight range of 12–15 g were used. After the transfer slugs were gently manually shaken in the buffer for 10 min and left to crawl out of the container, leaving the mucus solution at the bottom of the container. Thereafter, the slugs were rinsed with tap water, returned to their containers, fed, and left to recover. The obtained mucus solution was diluted with additional PBS to a total volume of 0.5 l and shaken on the rocker for 24 h at 4 °C. The mucus solution was then filtered using a series of 3 kitchen sieves (Zpts Kolbiarz) with decreasing mesh size (X = 1 × 1 mm, Y = 0.75 × 0,75 mm, Z = tightly woven filaments; Fig. 5a,e) to remove the stiff and insoluble fraction of the mucus which forms a rigid foamy cap on the top of the mucus solution within several minutes after collection (Fig. 5b–d). After filtering through the kitchen sieves only the fully liquified fraction was collected for centrifugation.

Differential centrifugation was carried out on Avanti JXN-26 equipped with a fixed angle rotor JLA 9, 1000 (Beckman Coulter). First, the mucus solution was centrifuged at 2000×*g* for 20 min at 4 °C and the supernatant was transferred to a clean centrifuge 500 ml bottle. Subsequently, the solution was centrifuged at 10,000×*g* for 30 min at 4 °C and at 15,000×*g* 4 °C for another 60 min. This EXs solution was filtered through a hydrophilic nylon net filter with a pore size of 30 μm (Merck). In the final step, the EXs were isolated from the solution by ultracentrifugation at 100,000×*g* for 2 h at 4 °C (Optima XPN-90, swinging bucket rotor SW32Ti, Beckman Coulter). The pellet was resuspended in a fresh PBS buffer and this step was repeated. The final isolate was resuspended in 1 ml of PBS with a complete inhibitor cocktail (Roche) and 5 μM Marimastat (Sigma-Aldrich) and stored at − 20 °C for further use.

### EXs characterisation

The size of isolated vesicles was characterised by the *DLS* analysis using the Zetasizer Nano-ZS instrument equipped with a 633 nm He–Ne laser and a 173° detection angle positioned detector (ZEN3600, Malvern Instruments). Samples of a constant volume of 120 μl were measured with a controlled temperature at 25 °C and an assumed refraction factor of 1.331 in disposable plastic micro cuvettes (ZEN0040, Malvern Panalytical). The data were analysed using the Malvern Panalytical software.

Vesicle size and concentration were analysed by *NTA* using NanoSight NS3000 (Malvern Instruments). Data collection and analysis was performed by the NTA software. Samples of approximately 300 μl were loaded into the flow-cell top-plate chamber. The chamber was illuminated by the 405 nm laser beam from the bottom, leading to the scattering of light by the particles in the sample solution. Each sample was analysed for 60 s, three times.

*TEM* was performed with tungsten filament (HT7820 Hitachi). For imaging, isolated frozen EXs (storage at − 20 °C) were thawed, mixed (1:1) with 4% paraformaldehyde (PFA) (w/v) in 0.1 M sodium phosphate buffer (PB) pH 7.4 and incubated in the fridge (4 °C) overnight. 5 µl of sample drops were deposited on parafilm and 200-mesh formvar/carbon grids (Merck) were placed on top of the drops and incubated for 30 min. Grids were then transferred on drops (100 µl) of PB for 1 min and then on drops (20 µl) of 1% glutaraldehyde (GA) in PB for 5 min. Grids were washed on drops (100 µl) of deionized water 8 times for 2 min. Finally, samples were stained on the drops (100 µl) of UranyLess stain for 3–5 min. The rest of UranyLess on the TEM grids was blotted with filter paper and grids were left to air dry for 1 h. Samples were observed in contrast mode with the accelerated voltage set to 100 kV and the beam current to 10 µA. Captured images were processed by the bitmap graphics software Gimp.

Exosomal protein quantification was performed using the *BCA protein assay* Kit (Merck) and *CBQCA protein quantitation assay* kit (Thermo Fisher Scientific). For the BCA assay 25 μl of each sample were mixed with 200 μl of working reagent, followed by heating at 37 °C for 30 min. EXs lysates were analysed, using 20 μl of each sample, 5 μl of RIPA 5× and 200 μl of working reagent and heated at 37 °C for 30 min. All samples were prepared in triplicates. A standard curve (0–2000 μg/ml) was prepared from six serial dilution points of bovine serum albumin (BSA) and working reagent, heated at 37 °C for 30 min. The CBQCA protein quantitation assay was performed according to the manufacturer protocol while using 1× PBS instead of the 0.1 M sodium borate buffer. The absorbance (at 562 nm for BCA assay) and fluorescence (excitation 465 nm, emission 550 nm for CBQCA assay) were measured in 96-well microplates (Thermo Fisher Scientific) using the GloMax Discover Microplate Reader (Promega). The data were analysed by GraphPad Prism (GraphPad Software).

For the *exosomal RNA isolation,* the exoRNeasy Midi Kit (Qiagen) was used. 500 µl of isolated EXs in PBS were mixed with 1 ml of lysate buffer and processed according to the manufacturer's protocol. The isolated RNA was mixed with 2× RNA Gel Loading Dye (Life Technologies) and analysed by gel electrophoresis using 1% agarose gel in 1× TBE buffer (0.13 M tris, 45 mM boric acid, 2.5 mM EDTA) stained with 10,000× diluted GelGreen Nucleic Acid Stain (Labmark) and recorded by the G:BOX gel doc system (Syngene). siRNA 21mer duplex with dTdT overhang (Sigma-Aldrich) was used as a loading control. Concentration was measured by a spectrophotometer DS-11 FX (DeNovix). The data were analysed by GraphPad Prism (GraphPad Software).

*Western blotting* was used to verify the presence of exosome markers (adhesion proteins) in the slug EXs. A lysis buffer composed of RIPA 1X, mercaptoethanol 5% and 5 mM PMSF was used to lysate exosome samples. Samples were incubated with lysis buffer for 30 min at 4 °C, followed by subjection to the SDS-PAGE on a 12% polyacrylamide gel. Proteins were blotted onto the nitrocellulose membrane. 5% milk in TBST was used as a blocking agent and for antibody dilution. The membrane was incubated with the primary antibody (diluted 500× in 5% milk in TBST) overnight at 4 °C. Subsequently, the membrane was washed six times in TBST and incubated with the secondary antibody at room temperature for 1 h. Washed membrane was incubated in SuperSignal West Femto Maximum Sensitivity Substrate (Thermo Fisher Scientific) and visualised. Primary antibodies used: Galectin-1/LGALS1 Antibody, Galectin-3/LGALS3 (D4I2R) XP Rabbit mAb, Recombinant Anti-Galectin 8/Gal-8 antibody (Abcam), and Galectin-9 (D9R4A) XP Rabbit mAb. Secondary antibody: Anti-rabbit IgG, HRP-linked Antibody. All antibodies except Galectin-8 were purchased from Cell Signaling Technology.

### EXs cargo loading and cell internalisation assays

The procedure of *EXs loading by DOX* was adapted from^71^ and modified as follows (Fig. 3). The 5 μl of DOX solution (1000 µg/ml) was added to 50 μl exosomes (300 μg/ml of total proteins as measured by BCA) in PBS and incubated at room temperature for 2 h. To determine the DOX uptake by the exosomes, the mixture was ultracentrifuged for 30 min at 100,000×*g*, the supernatant with the free DOX was discarded, and the EXs were washed and resuspended in 1 ml PBS. This step was repeated two times to effectively remove the residual free DOX (Fig. 3). The concentration of DOX in the EXs was determined by LC–MS/MS.

For the *LC–MS/MS analysis*, separation was carried out using an Agilent 1290 Infinity II UHPLC system (Agilent Technologies) with a Kinetex Polar C18 analytical column 2.1 × 150 mm, 2.6 µm (Phenomenex) at a flow rate of 0.4 ml/min. The mobile phases consisted of (A) H_2_O with 0.1% HCOOH and (B) acetonitrile with 0.1% HCOOH. The HPLC system was coupled to an Agilent G6495A Triple Quadrupole mass spectrometer equipped with an Agilent Jet Stream electrospray ionisation source. Standard curves of signal response vs concentration for the quantification of DOX were created via plotting signal response to the range of 0–10 µg/l DOX in PBS. The influence of the matrix on DOX determination was verified by spiking each sample with 1 µg/l DOX.

*The U87 human glioblastoma cell line* (ATCC) was maintained in complete eagle minimal medium supplemented with 100 mM nonessential amino acids (NEAA), 10% foetal bovine serum, 2 mM glutamine, 100 µl penicillin, and 0.1 mg streptomycin (Thermo Fisher Scientific). The cells were cultivated at 37 °C in 5% CO_2_.

*The Tobacco Bright Yellow-2* (BY-2) suspension culture of *Nicotiana tabacum* was provided by the Institute of Experimental Botany of the Czech Academy of Science. Cells were cultivated in Erlenmeyer flasks in the dark at 26 °C on a shaker (105 RPM). Media were prepared using 4.3 g/l Murashige-Skoog basal salt; 30 g/l saccharose; 0.2 g/l KH_2_PO_4_; 0.1 g/l inositol; 1 mg/l thiamine and 0.2 mg/l 2,4-dichlorophenoxyacetic acid. The pH of the media was adjusted to 5.6–5.8 and it was sterilised in an autoclave at 121 °C for 20 min. BY-2 cells for experiments were collected a week after their transfer into fresh media.

We performed an *EXs internalisation assay* in order to evaluate the cellular uptake of slug EXs. U87 (human glioblastoma) and BY-2 (plant) cells were incubated with BODIPY TR Ceramide (Thermo Fisher Scientific) stained EXs for 4 h. 1 × 10^8^ EXs in 100 µl of PBS were incubated with 2 µl of BODIPY TR Ceramide as per the provider datasheet. Subsequently, the labelled EXs were purified by Exosome spin columns and incubated with cells. Untreated cells were used as a negative control. The uptake of EXs was evaluated by an HC PL APO CS2 20 × 0.75 DRY objective of the CLSM SP8microscope (Leica) with 488 nm excitation and 650–750 nm emission (red channel).

### Ethics approval

The Jan Evangelista Purkyně University is a certified facility for the use of animals in research (Veterinary approval number CZ 42760032, Ministry of Agriculture of the Czech Republic approval number MZE-19331/2022-13143). ML, OJ are certified for planning and performing experiments on animals (certificate numbers: ML-CZ 03236, OJ-CZ 02834). No animals were harmed during experimental procedures (mucus collection).

## Supplementary Information

Supplementary Figure S1.

## Data Availability

All raw data and original western blot images are available upon request by the corresponding author.
