# Supplementary material for: Mucus-derived exosome-like vesicles from the Spanish slug (Arion vulgaris): taking advantage of invasive pest species in biotechnology
Source: Sci Rep. 2022 Dec 16;12:21768. doi: 10.1038/s41598-022-26335-3 (PMC9870906; doi:10.1038/s41598-022-26335-3)
Supplement: Supplementary file 1 — Supplementary Figure S1. [file 41598_2022_26335_MOESM1_ESM.docx]

**SUPPLEMENTAL INFORMATION**

**Mucus-derived exosome-like vesicles from the Spanish slug (Arion vulgaris): Taking advantage of invasive pest species in biotechnology**

Michaela Liegertová, Alena Semerádtová, Michaela Kocholatá, Michaela Průšová, Lenka Němcová, Marcel Štofik, Sylvie Kříženecká, Jan Malý, Olga Janoušková


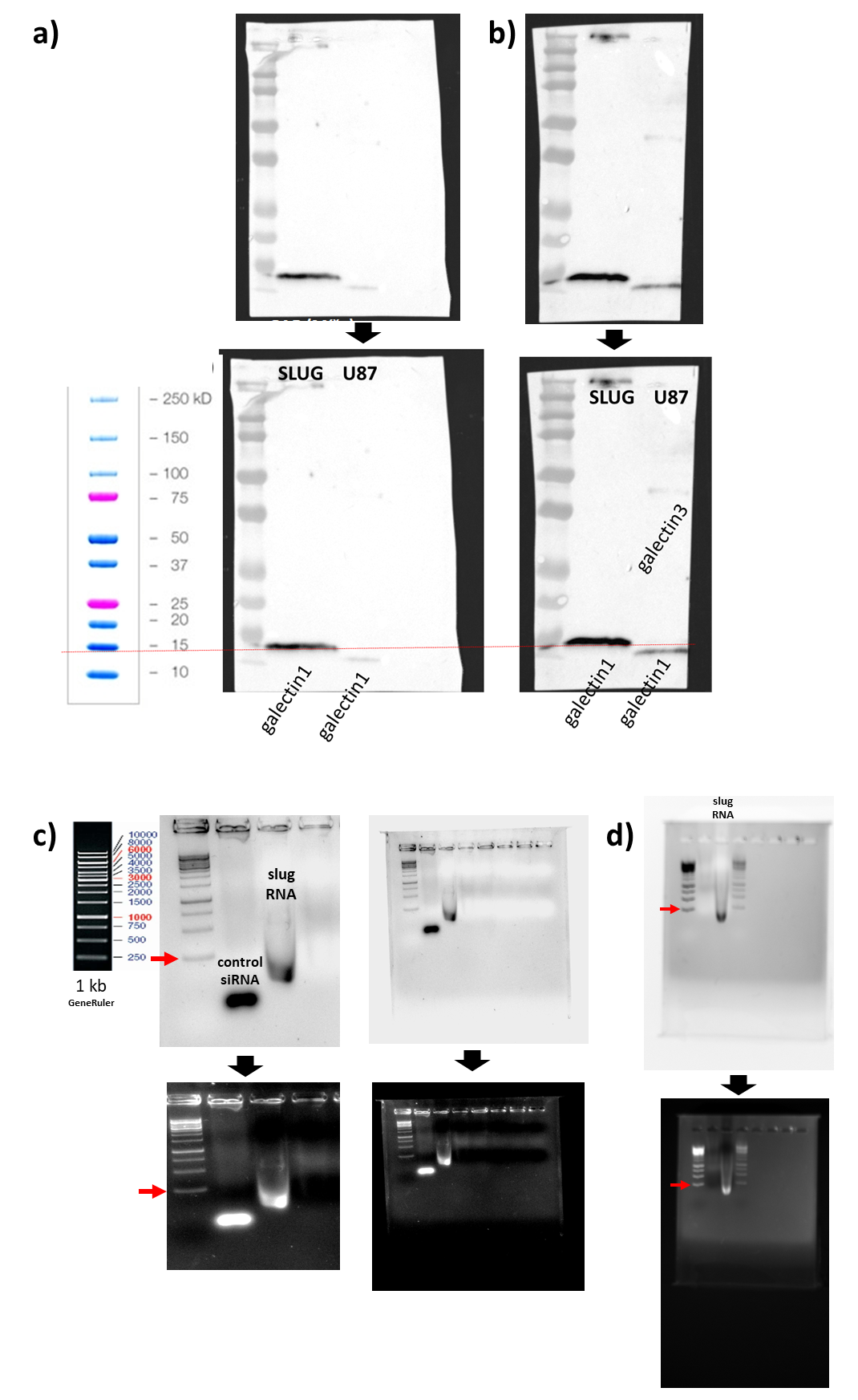


**Figure S1: Original western blot and gel images**

**(a-b)** Original uncropped images of western blot membranes from two separate isolations of slug exosomes (lane 1) and U87 human glioblastoma cell line (lane 2; used as positive control) incubated with 500 x diluted Galectin-1/LGALS1 Antibody **(a)**, and a mixture of 500 x diluted Galectin-1/LGALS1 Antibody and Galectin-3/LGALS3 **(b).** The Precision Plus Protein Dual Color Standards (Bio-Rad) was used as a ladder. **(c-d)** Cropped and uncropped images of the gels from two separate RNA isolation procedures. The GeneRuler 1 kb DNA Ladder (Thermo Scientific) was used as a ladder; red arrows indicate the 250 bp band. **(c)** Slug RNA (lane 3) compared to control siRNA used as positive control (lane 2); gel was used to generate Figure 2c. All four photographs belong to the same gel. **(d)** Uncropped image of gel from slug RNA isolation (lane 3). Both images belong to the same gel.
